# Role of B-Cell Lymphoma/Leukemia 11A in Normal and Malignant Hematopoiesis

**DOI:** 10.3390/biology14010026

**Published:** 2025-01-01

**Authors:** Haihang Zhang, Junhao Zeng, Fangling Zhang, Jing Liu, Long Liang

**Affiliations:** 1 Department of Hematology, the Second Xiangya Hospital, School of Life Sciences, Hunan Province Key Laboratory of Basic and Applied Hematology, Central South University, Changsha 410011, China; z1138645288@163.com (H.Z.); 222511067@csu.edu.cn (F.Z.); 2 Xiangya School of Medicine, Central South University, Changsha 410013, China; doctor_zjh@csu.edu.cn

**Keywords:** B-cell lymphoma/leukemia 11A, normal and malignant hematopoiesis, potential clinical application, gene editing, medicine

## Abstract

B-cell lymphoma/leukemia 11A (BCL11A) is a crucial transcriptional regulator, widely recognized for its role in controlling fetal hemoglobin and its potential as a gene therapy target for inherited hemoglobinopathies. Beyond this, recent studies have also highlighted its key role in the maturation and function of immune cells and erythrocytes, mediated through the regulation of various molecules during hematopoietic development. The dysregulation of BCL11A disrupts downstream molecular pathways, contributing to the development of several hematological malignancies, particularly leukemias. This review provides a comprehensive overview of the role of BCL11A in normal and malignant hematopoiesis, details the hematological disorders associated with its dysregulation and explores the current therapeutic strategies targeting this transcription factor.

## Simple Summary

The transcriptional regulation of BCL11A shows stage-specific characteristics and plays an important role in the development of hematopoietic lineages, which is closely related to the occurrence of blood cell diseases. We aim to summarize the functions of BCL11A within the hematopoietic system and related diseases and discuss its feasibility as a therapeutic target for the treatment of hematological disorders.

## 1. Introduction

Transcription factors regulate gene transcription by binding to specific regions of DNA and play a pivotal role in maintaining the self-renewal, fate determination, differentiation and maturation of hematopoietic stem cells. A number of well-characterized transcription factors have been identified, including SCL/TAL-1, which plays a pivotal role in maintaining stem cell characteristics; GATA-1, which is instrumental in controlling the differentiation of erythroid cells [1]; PU.1, which affects the generation of lymphocytes and myeloid cells [2]; and RUNX1, which is crucial in regulating the generation of leukocytes in the bone marrow [3]. Transcription factors play an important role in both normal hematopoiesis and the pathogenesis of hematological diseases.

In recent years, technological advancements, particularly the application of CRISPR/Cas9 in disease treatment, have highlighted the potential significance of the transcription factor BCL11A (B-cell lymphoma/leukemia 11A) as a promising target for further investigation. This transcription factor has been demonstrated to be closely linked to fetal hemoglobin levels in whole-genome association analyses [4]. A reduction in BCL11A expression has been observed to result in the upregulation of HbF, thereby offering a potential avenue for therapeutic intervention in the treatment of beta-hemoglobin disorders [5]. Furthermore, recent studies have identified BCL11A as a critical factor in the development of the hematopoietic system. This review article aims to elucidate the significant roles and mechanisms of BCL11A in the erythroid lineage, the lymphocyte lineage (particularly B cells) and the broader hematopoietic cell lineage. Additionally, it reviews the various blood disorders resulting from abnormal BCL11A activity and the current therapeutic approaches targeting these conditions.

## 2. Expression and Structure of the BCL11A Gene

**Figure 1:**
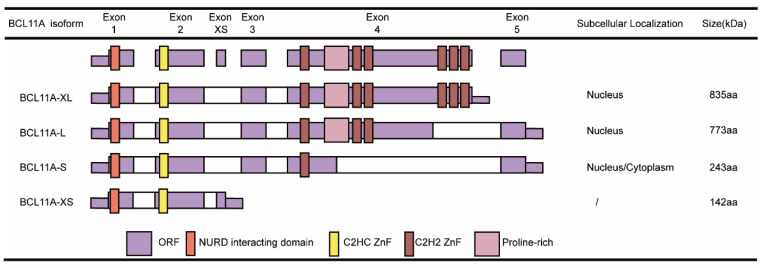
Schematic representation of functional domains, protein subcellular localization and number of nucleotides for BCL11A.

BCL11A and BCL11B are two members of the BCL11 zinc-finger transcription factor family that were first identified and found to interact with chicken ovalbumin upstream promoter transcription factor (COUP-TF), leading to them being named COUP-TF-interacting protein 1 and COUP-TF-interacting protein 2 [6]. Human BCL11A contains 15 exons and encodes at least four transcripts (BCL11A-XL, -L, -S and -XS) (Figure 1). The main difference between these four transcripts is in exons 3 and 4. The longest BCL11A-XL contains the complete exons 3 and 4, while selective cutting of the fourth exon yields BCL11A-L and BCL11A-S, and BCL11A-XS (1.5 kb/25 kD) only contains exons 1 and 2 [7].

BCL11A-L has four zinc fingers, and two zinc fingers at the C-terminal recognize and bind to the 5′-GGCCGG-3′ DNA-binding motif [8]. Because BCL11A-S lacks a C-terminal zinc finger, it does not recognize DNA target sequences but acts as an endogenous antagonist of BCL11A-L, preventing homo-oligomerization [9]. Studies have found that the S isoform with the same N-terminus resulted in greater transcriptional repression than the XL and L isoforms [7]. BCL11A-XL is the most abundant isoform identified in human lymphoid samples; it contains six homologous Krüppel C2H2 zinc fingers and proline-rich domains and acidic domains between the zinc fingers [10]. There are data showing that the BCL11A-XL subtype is strongly associated with low γ-globin production and high β-globin gene expression [8].

BCL11A is predominantly expressed in the brain and in the majority of hematopoietic cells, including hematopoietic stem cells, common lymphoid progenitors, B cells and early T-cell progenitors. It is noteworthy that its expression is relatively weak in T lymphocytes. Each isoform of BCL11A exhibits a distinct expression pattern. For example, BCL11A-XL is selectively expressed in normal B cells, while BCL11A-S is found in B-cell malignancies. In both human and rat brains, BCL11A-S is widely distributed, whereas BCL11A-L is predominantly expressed in the cerebral cortex. The implications of these diverse expression patterns of BCL11A in disease regulation remain unclear and warrant further investigation.

## 3. The Role of BCL11A in Hematopoietic Stem Cells

**Figure 2:**
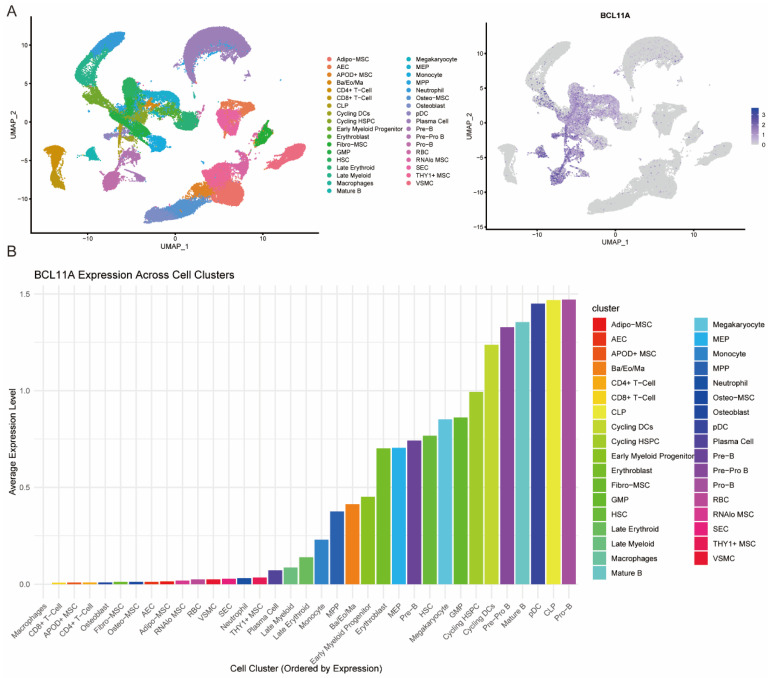
Gene expression analysis of BCL11A in normal human hematopoiesis. The data were analyzed as previously described [19]. (**A**) Annotation clustering of the single-cell dataset GSE253355 for normal human bone marrow (**left**) and feature plot of BCL11A expression in each cell population (**right**). (**B**) Boxplot of the average expression of BCL11A in each cell population. The cells are abbreviated as follows: arterial endothelial cell (AEC), sinusoidal endothelial cell (SEC), vascular smooth muscle cell (VSMC), basophil (Ba), eosinophil (Eo), mast cell (Ma), red blood cell (RBC), plasmacytoid dendritic cell (pDC), common lymphoid progenitor (CLP), megakaryocyte erythroid progenitor (MEP), granulocyte monocyte progenitor (GMP), multipotent progenitor (MPP), hematopoietic stem and progenitor cell (HSPC), hematopoietic stem cell (HSC), megakaryocyte/erythroid (Meg/E) and mesenchymal stromal cell (MSC).

**Figure 3:**
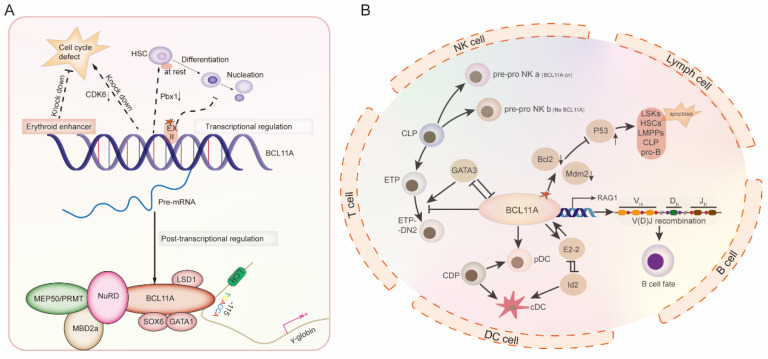
Schematic diagram of transcriptional and post-transcriptional function of BCL11A in normal hematopoiesis. (**A**) The role of BCL11A in HSCs, erythroid development and the regulation of fetal hemoglobin. (**B**) The role of BCL11A in lymphocytes, B cells, T cells, DC cells and NK cells.

Hematopoiesis is a strictly controlled process in which hematopoietic stem cells (HSCs) and progenitor cells grow and differentiate into various cell lines. Hematopoiesis is a tightly controlled process in which hematopoietic stem cells maintain the function of the hematological system by self-renewing and differentiating into different types of blood cells in the bone marrow. BCL11A is expressed during different hematopoietic processes (Figure 2A,B) and is widely involved in the development of multiple lineages (Figure 3A,B), including HSC, erythrocyte and lymphocyte lineages. It directly or indirectly regulates the expression of multiple hematopoietic lineage-related genes via transcriptional inhibition/activation and complex formation.

A recent report has suggested that BCL11A may be expressed during the pre-HSC stage [11]. Some data suggest a significant contribution of BCL11A in maintaining HSC quiescence [12]. In HSCs, BCL11A deletion leads to the downregulation of the expression of the HSC static maintenance gene Pbx1 [13]. Similarly, the loss of BCL11A causes HSCs to exhibit aging-like cell cycle defects, which may be related to CDK6 downregulation [14]. In addition, in mouse embryonic development studies, *BCL11A* deletion has been shown to lead to reduced phenotypic HSC, suggesting that BCL11A is essential for HSC development [14]. However, the deletion of *BCL11A* in human HSC CD34+ cells did not cause global changes in the differentiation state of the cells; the expression of multiple important interacting erythroid transcription factors, such as NF-E2, GATA1 and FOG1, did not change [15]; and bone marrow development was not significantly affected by *BCL11A* deletion. In a tetracycline-inducible BCL11A knockout mouse model, the ablation of BCL11A resulted in the suppression of B-cell development in the bone marrow, while other lineages were unaffected. Furthermore, B cells in tissues outside the bone marrow were also not impacted [16]. It is noteworthy that, despite *BCL11A* not being a prerequisite for erythropoiesis, *BCL11A* knockdown has been demonstrated to significantly impair the long-term engraftment and cell cycle regulation of HSCs during mouse bone marrow transplantation [17]. This defect can be reversed through the erythroid-specific knockdown of *BCL11A* or the *BCL11A* erythroid enhancer [17,18]. This suggests that BCL11A plays an important role in HSC engraftment and the reconstitution of the blood system. However, the mechanism by which this occurs remains to be elucidated.

It has been demonstrated that BCL11A is highly expressed in hematopoietic stem cells (HSCs) and is also expressed in granulocyte–macrophage progenitors (GMPs), pre-pro-B cells, pro-B cells, common lymphoid progenitors (CLPs) and other cells, as well as in other stages following lineage differentiation [19] (Figure 2). The following sections of this article will review the specific functions of BCL11A in the development of different lineages.

## 4. BCL11A Essential for Lymphoid Lineage Development

As it is essential for normal lymphocyte development in humans, BCL11A is highly expressed in various lymphoid stem cells, B cells and T cells. Mouse embryos transplanted with a *Bcl11a* mutant missing exon 1, which codes for translation initiation sites and the first 18 amino acids, had B-cell defects and exhibited the abnormal development of T-cell subtypes [20]. BCL11A affects B-cell development by acting on a variety of B-cell regulatory genes, including *Rag1*, *Pax5*, *Il7r*, *Cd19*, *Ebf1* and *Vpreb2*, which are expressed in the early and late B-cell stages [20]. It has been reported that BCL11A directly binds to the *RAG1* promoter, activates *RAG1* and *RAG2* transcription and regulates B-cell fate by controlling VDJ recombination [21]. EBF1-binding sites were found in the *Bcl11a* locus, and the expression of *Bcl11a* was affected by EBF1, which explains why Bcl11a regulates B-cell development [20,22]. The deletion of *BCL11A* restricts the proliferation of B-cell precursors and increases apoptosis by negatively regulating the expression of P53 [16,23]. The expression of BCL11A is significantly increased in patients with malignant B lymphoblastoma, providing a possible explanation for the resistance to apoptosis in patients with chronic lymphocytic leukemia [7,24]. Different isotypes of BCL11A have different distributions in B cells, with BCL11A-XL preferentially distributed in normal B cells and BCL11A-S preferentially distributed in malignant B cells. Further research is required to clarify the distribution of different components of BCL11A for the development of B-cell diseases and to provide new insights into the pathogenesis and development of diseases [10]. In general, BCL11A is particularly important for the development of lymphoid cells represented by B cells, which also determines its indirect regulatory effect on the immune system; thus, BCL11A is necessary for the normal development of individuals.

Multiple lines of evidence suggest that BCL11A is equally important for T-cell development. The loss of *BCL11A* can eliminate the potential of HSCs to differentiate into T cells for lymphatic development [16]. Studies have shown dynamic changes in BCL11A during T-cell development. During T-cell development, BCL11A is expressed in almost all early T-cell progenitor cells. During T-cell commitment, the expression decreases sharply, whereas the expression of BCL11B increases significantly, suggesting that thymus cells can be distinguished before and after T-cell commitment [16]. However, the mechanism by which BCL11A and BCL11B coordinate T-cell commitment requires further study. In addition, during T-cell lineage differentiation, BCL11A appears to antagonize the T-cell-specific gene GATA3, delaying the development of the T-cell lineage; transcription factor PU.1 (SPI1), which is highly expressed in the earliest intrathymic T-cell progenitors but must be downregulated during T-lineage commitment, has a similar effect. The destruction of SPI1 and BCL11A leads further along the T-cell developmental trajectory during early T-cell progenitor progression to double-negative 2a (DN2a) (DN:CD4-CD8-), which also suggests that they play a role in controlling cell development [25].

BCL11A mainly regulates the fate of B and T cells in the lymphoid system. Beyond this, it is worth noting that some studies have shown that, during T-cell commitment, the BCL11A expression decreases sharply, whereas the expression of BCL11B increases significantly, suggesting that BCL11A and BCL11B may play opposite roles in the development of T cells, but the specific mechanisms are unknown [16]. At the same time, different subtypes of BCL11A may be associated with different types of lymphomas, but there have been few studies to date. Further investigation is needed.

## 5. Different Role of BCL11A in Dendritic Cell Subtypes

Dendritic cells differentiate from the monocyte/macrophage lineage or from the common dendritic cell progenitors (CDPs), which are a heterogeneous group of cells. CDPs can partially develop from myeloid lineage cells or lymphoid lineage cells, including two major subsets, plasmacytoid dendritic cells (pDCs) and conventional dendritic cells (cDCs), which are vital for coordinated and adaptive immunity. BCL11A is required for CDP development and regulates the expression of the IL-7 receptor and FLT3 [26]. The FLT3 ligand is essential for dendritic cell development [27]. Defects in BCL11A lead to the selective loss of CDP cells and further impair pDC and cDC generation [26]. The transcription factor E2-2 partially maintains the cell fate of mature pDCs through BCL11A and opposes the “default” cDC fate [28]. However, BCL11A is not necessary for cDC development in vivo, and studies in BCL11A-deficient mice have shown that cDCs persist when pDCs are abolished [26]; the high expression of BCL11A in pDC cells provides a reasonable explanation [29]. Therefore, the expression of BCL11A is also used to distinguish between human and mouse pDC and cDC dendritic lineages [30]. There is a basic need for BCL11A during pDC development; BCL11A regulates the transcription of the pDC-essential gene E2-2 and pDC differentiation regulators ID2 and MTG16 [31], thereby regulating the generation of fetal and adult pDCs. In the gene expression analysis of CDPs, it was found that pDC factors (E2-2, BCL11A and RUNX2) were highly expressed but cDC factors (ID2, BATF3 and BCL6) were not, which seemed to support the default CDP pDC differentiation model [32]. In summary, BCL11A plays an important role in the development of the dendritic cell lineage, particularly in pDCs.

## 6. BCL11A Controls the Hemoglobin Switch in Erythroid Lineage

Erythroid development includes the process from the expansion of erythroid progenitor cells to the terminal differentiation of erythrocytes, the formation of reticulocytes and their further maturation into erythrocytes [33]. BCL11A is highly expressed in late-stage erythroid cells, which is mainly related to their hemoglobin expression. The targeted destruction of BCL11A exon 2 using zinc-finger nucleases has been reported to reduce erythropoiesis and significantly reduce the normal denucleation efficiency of erythroid cells [34]. However, the RNA-binding protein LIN28B negatively regulates BCL11A protein synthesis and has no significant effect on erythroid differentiation [35]. Similarly, recent genome-wide data have shown that patients with BCL11A haploinsufficiency do not have abnormal erythropoiesis [36]. The function of BCL11A in the erythroid lineage may be closely related to its protein abundance, and its specific mechanism of function needs to be further elucidated.

Along with its role in erythroid development, BCL11A is known as a γ-globin gene-silencing factor, originally identified in a genome-wide association study [37]. Several studies have shown that additional transcription factors and coregulators that directly or indirectly affect the activity of BCL11A or its expression influence the regulation of HBG1/2 genes (Figure 3A). The regulation of BCL11A is mainly at the transcriptional level. The transcription factors ATF4, KLF1, NFIA/X, GATA1 and Mi2β (members of the NuRD complex) are involved in controlling the transcriptional expression of BCL11A, while the transcription factor HIC2 inhibits BCL11A transcription [38,39,40,41,42]. Among them, ATF4 directly stimulates the transcription of BCL11A by binding to its enhancer and fostering enhancer–promoter contact [38]. KLF1 binds to the CACCC box in the mouse Bcl11a promoter in adult bone marrow erythroid cells and plays a regulatory role [39]. NFIA and NFIX bind strongly at BCL11A’s intronic enhancer regions, suggesting that NFI proteins might modulate BCL11A expression [40]. Mi2β is enriched in the proximal promoter regions of the BCL11A and KLF1 genes in erythroid cells and promotes the expression of these genes [41]. Genetic studies have identified a restricted 14kb region in BCL11A intron 2 as being highly associated with HbF levels; GATA-1 enrichment and acetylated histone H3 were found in this region, suggesting the presence of a regulatory element responsible for the regulation of BCL11A [37].

The unique structure of BCL11A determines its unique functionality; BCL11A’s N-terminal contains a NuRD-complex-binding peptide that plays an important role in the inhibition of γ-globin transcription [5,41]. Downregulation of the expression of NuRD subunits, such as CHD4 [43], MBD2 [44] and HDAC 1/2 [45], induces HbF expression. The direct interaction between the MBD2a–NuRD model and BCL11A recruits MEP50/PRTM5 to form a stable large complex, localizes methylated proximal HBG promoters and inhibits HBG transcription [46]. In addition, lysine-specific demethylase 1 (LSD1) and SRY-Box transcription factor 6 (SOX6) have been shown to interact and cooperate with BCL11A for the transcriptional silencing of γ-globin [47,48]. The zinc fingers of BCL11A play an important role in the inhibition of γ-globin transcription. Three C-terminal C2H2 zinc fingers directly bind to the distal TGACCA motif in the -115 HPFH region of the γ-globin promoter while also binding to SOX6 [49]. Owing to the “undruggable” nature of the BCL11A transcription factor and its important physiological function, the inhibition of BCL11A for the reactivation of HbF has become a challenge. Common genetic variants of BCL11A associated with HbF levels have been found in noncoding sequences modified by erythrocyte-specific enhancers [50]. The destruction of the erythrocyte-specific enhancer +58 BCL11A effectively reduced the expression of BCL11A and increased the HbF levels, as demonstrated in patients with beta-thalassemia and sickle cell disease (SCD) [17,51].

## 7. Hematological Diseases

BCL11A is a key factor in the development and normal functioning of the hematopoietic system. Studies have shown that BCL11A plays a vital role in hematological diseases, particularly hematological malignancies (Table 1). BCL11A is highly expressed or mutated in NK-/T-cell lymphoma [52,53], acute myeloid leukemia [54,55], childhood acute leukemia [56], chronic lymphocytic leukemia [57], acute lymphoblastic leukemia (ALL) [58] and aggressive B-cell lymphoma [59] and is associated with poor prognosis; its downregulation may predict complete remission in patients with B-cell ALL [16,23]. However, in juvenile myelomonocytic leukemia with elevated Hbf, although BCL11A is important in Hbf regulation, it has not been found to be related to its pathogenesis [60].

In addition to identifying BCL11A as a key gene in these hematological diseases, scientists have investigated its interaction with other key factors in the pathogenesis of these diseases. P53 is a well-known tumor suppressor gene [61], and BCL11A inhibits BCL2, BCL6, MDM2 and SIRT1, which can upregulate P53 expression [62,63,64,65,66,67,68]. PU.1 plays a non-redundant role in the development of the myeloid cell lineage and is considered a master regulator of macrophage differentiation [69,70,71]. BCL11A inhibits the target genes of PU.1, such as ASB2, CLEC5A and FCGR3, and is involved in the occurrence and development of malignant human bone marrow tumors [72]. BCL11A also inhibits the tumor suppressor gene P21 and disrupts the cell cycle [73].

In classical Hodgkin lymphoma, non-Hodgkin lymphoma and diffuse large B-cell lymphoma, BCL11A is found to be coamplified with the proto-oncogene REL and is associated with enhanced c-Rel expression [10,74]. The activation of another proto-oncogene, the RAS oncogene, is functionally equivalent to NF1 deficiency, and BCL11A can promote the proliferation and growth of NF1-deficient cells and jointly play an oncogenic role [73,75].

BCL11A is associated with t(2;14) chromosomal translocations in B-cell chronic lymphoid leukemia, and the juxtaposition of the immunoglobulin heavy-chain enhancer results in the overexpression of BCL11A mRNA [76]. In acute myeloid leukemia, high BCL11A expression is associated with a poor response to chemotherapy and promotes the proliferation and engraftment of TRIB1-expressing acute myeloid leukemia cells in vitro and in vivo [54,72]. In pediatric ALL, patients with mixed-lineage leukemia rearrangement have a poor prognosis, and the upregulation of BCL11A expression may lead to mixed-lineage leukemia rearrangement ALL [58]. In acute-phase chronic myeloid leukemia, BCL11A may play a role in the transition from the chronic to the acute phase [55]. Translocations of BCL11A and Ig heavy chain sites have also been reported in B-cell malignancies [77], suggesting that BCL11A directly or indirectly promotes pathogenesis and is one of the targets guiding clinical treatment strategies.

**Table 1:** BCL11A in malignant hematopoietic diseases and its functions.

Disease	Involved Molecules or Pathways	Function
Natural killer/T-cell lymphoma (NKTCL)	/	BCL11A is one of the most recurrently mutated genes [53]
	RUNX3, c-MYC, and P53; RUNX3–MYC pathway	High BCL11A expression is an independent unfavorable prognostic factor for OS and PFS among NKTL patients [78]
Acute myeloid leukemia (AML)	MDR1	AML patients with high BCL11A and MDR1 expression had the lowest complete remission and highest relapse rate [54]
Chronic lymphocytic leukemia/small lymphocytic lymphoma (CLL/SLL)	IgV (H)	CLL/SLL with BCL11A/IgH rearrangement is characterized by atypical morphologic features and unmutated IgV (H) genes [76]
Aggressive B-cell chronic lymphocytic leukemia (CLL)/immunocytoma	REL	Clustered breakpoints occurred in the CpG island associated with BCL11A [79]
Extranodal B-cell non-Hodgkin lymphoma (B-NHL) Hodgkin’s disease (HD)	REL	BCL11A was coamplified with REL in B-NHL cases and HD lymphoma cell lines [79]
Chronic lymphocytic leukemia (CLL)	Serum lactate dehydrogenase; Binet stage A and mutated IGHV	CLL patients with high expression of BCL11A may tend to have a better prognosis [57]
	BCL2 and BCL3	Concurrent rearrangements of BCL2, BCL3, and BCL11A genes in atypical chronic lymphocytic leukemia [80]
Acute myeloid leukemia (AML)	Trib1 and PU.1	Promoting the proliferation and engraftment of Trib1-expressing AML cells in vitro and in vivo; inhibiting the growth of HL-60 cells and their engraftment to the bone marrow [72]
	p21Cip1	Causing leukemia in the absence of Nf1 in mice [73]
Chronic myeloid leukemia (CML)	/	Operating in the transformation of CML from the chronic to acute phase in some people [55]
Juvenile myelomonocytic leukemia (JMML)	/	BCL11A gene locus is not subject to changes in CpG island methylation [60]
Acute lymphoblastic leukemia (ALL)	MLL rearrangement (MLL-r)	Upregulated BCL11A gene expression in childhood ALL may lead to MLL-r ALL development [58]
B-cell acute lymphoblastic leukemia (B-ALL)	Mdm2 and Pten	Bcl11a expression level was positively correlated with the Mdm2 and Pten relative expression levels in B-ALL patients [23]
T-cell leukemia	/	Mice transplanted with Bcl11a-deficient cells died from T-cell leukemia derived from the host [20]
Diffuse large B-cell lymphoma (DLBCL)	C-REL	[74]
Promyelocytic leukemia protein (PML)	STRT1 and P53	Interact directly with SIRT1, leading to transcriptional repression [67]

As a transcriptional regulator, BCL11A can play a role in hematopoietic diseases through its own mutation or affect other related blood regulatory factors and changes in oncogenes. Its roles and functions in various hematopoietic diseases are summarized below.

## 8. Potential Clinical Application

Because it has been confirmed that BCL11A plays an important role in the silencing of γ-globin, many scientists have focused on restoring γ-globin expression by eliminating or downregulating *BCL11A* expression to treat β-hemoglobinopathies such as β-thalassemia and SCD.

## 9. Targeting BCL11A to Switch Hemoglobin Through Gene Editing

Direct knockout of the BCL11A gene seems to be the most straightforward way to achieve this process, and many experimental results have demonstrated its effectiveness [5,15,81,82]. Because BCL11A is an indispensable regulator of the growth and development of the blood and nervous systems, the risks posed by the direct knockout or deletion of BCL11A cannot be ignored [14,16,31,36,83,84,85,86]; however, a few studies have suggested that the haplotype of BCL11A does not affect human immune function [87]. As an alternative, silencing BCL11A expression using siRNA and targeting BCL11A mRNA using short hairpin RNA have shown improved potential to induce γ-globin [5,88,89,90].

The safety of the knockdown of the erythroid enhancer of Bcl11a has been confirmed in several studies [91,92,93]; however, its effectiveness remains controversial. Although many studies have used different techniques to effectively downregulate Bcl11a expression and activate γ-globin expression in different cell lines by knocking out the erythroid enhancer of Bcl11a [51,83,94,95,96,97], the effect may vary across different cell lines. For example, CRISPR-Cas9 was used to knock out the BCL11A erythroid enhancer (including the GATAA motif) in the KU-812, KG-1 and K562 cell lines; γ-globin was found to be highly expressed in K562 cells but not in KU-812 or KG-1 cells [98].

CRISPR-Cas9 and lentivirus-mediated gene editing are common gene-editing methods; the disruption of the binding site for GATA1 at the BCL11A erythroid enhancer has been shown, in vitro and in vivo, to induce γ-globin expression [99]. In clinical studies, it was found that, in transfusion-dependent thalassemia patients, administering autologous CD34+ cells edited with CRISPR-Cas9 targeting the *BCL11A* erythroid-specific enhancer resulted in transfusion independence, with a mean total hemoglobin level of 13.1 g/per deciliter, and adverse effects such as headache and acute respiratory distress syndrome were observed in some of the patients [100]. Additional studies have shown that CTX001 effectively increased hemoglobin levels in patients with transfusion-dependent beta-thalassemia and SCD [96]. Exagamglogene autotemcel therapy (formerly known as CTX001) has been approved by the FDA for transfusion-dependent β-thalassemia and SCD; however, with reference to the example of elivaldogene autotemcel gene therapy consisting of autologous CD34+ cells leading to the development of hematological cancers in seven patients [101], for BCL11A erythroid enhancer editing, there may be a risk of secondary hematological malignancies in patients with transfusion-dependent thalassemia. The long-term supervision of patients is therefore required, and high costs are incurred [98]. In addition, editing schemes targeting BCL11A erythroid enhancers, such as zinc-finger nuclease-mediated editing, have been demonstrated not to negatively affect the survival or long-term proliferation of hematopoietic stem/progenitor cells and other lineages in vivo [83,91]. By targeting the BCL11A-binding site through nuclease-induced indels, base editing and HbF induction can be effectively achieved; however, this may also lead to large deletions between the HBG1 and HBG2 promoters and the loss of the HBG2 gene [93].

In addition to the erythroid enhancer of BCL11A, the BCL11A-binding site and BCL11A exon 2 in the HBG1/2 promoter are also the focus of gene therapy research. Several studies have shown that the presence of >G mutation pairs in the HBG1/2 promoter with -114C>T, -115C>T and -116A base editing; -117G>A, -114C>A, -114C>T and -114C>G 13 bp deletion (Δ13bp); and the introduction of -113A>G, -114C>T, -117G>C and -195C>G homologous recombination disrupt BCL11A binding [102,103,104,105,106,107,108]. The disruption of exon 2 of BCL11A by a zinc-finger nuclease resulted in a significant increase in the frequency of HbF+ cells and γ-globin levels [83].

Although editing at a single locus has been reported to have the potential to realize long-term stable γ-globin induction [15], the dual editing of cells does not impair engraftment, lineage differentiation or proliferation after transplantation into immunodeficient mice [109]. Therefore, many scientists are working on multiple editing and multiple interventions to achieve efficient and stable γ-globin induction. Examples of successful multiple editing or multiple interventions include the CRISPR/Cas9-mediated co-editing of HBG-113 and BCL11A erythroid enhancer [109], the CRISPR/Cas9-mediated combined knockout of BCL11A erythroid enhancer and the γ-globin promoter [92], the combined base editing of BCL11A erythroid enhancer and the correction of the HBB−28A>G promoter mutation [110] and the lentivirus mediation of short hairpin miR induced by BCL11A and ZNF410 [111]. All the aforementioned studies reveal the advantage of multiple editing in improving the induction efficiency. However, the safety of multiple editing is questionable because it is likely to induce tumors [109].

## 10. Targeting BCL11A Using Chemical Compounds

Medical intervention to downregulate BCL11A expression and then reactivate γ-globin expression is another frontier in the treatment of related hematological diseases; however, it has not been smoothly implemented. Most medicines studied in this manner have not been approved for marketing. Until 2018, hydroxyurea (HU) was the only SCD drug approved by the United States Food and Drug Administration (FDA) [112]. Until 2021, there was no FDA-approved HbF inducer for the treatment of β-thalassemia [113].

Research on HU has reached a relatively mature stage, and its mechanism of action and application limitations have been studied extensively. The use of HU results in the differential expression of miR-148b-3p, miR-32-5p, miR-340-5p and miR-29c-3p related to BCL11A expression [114], and its therapeutic effect is related to single-nucleotide polymorphisms of intron 2 of BCL11A: rs766432, rs11886868, rs4671393 and rs7557939 [52,94]. γ-Globin induction is improved when HU is combined with SRT2104 and SRT1720, which are activators of the BCL11A expression inhibitor SIRT1 [66,115]. The use of HU is also limited. It is effective for only approximately half of patients with SCD and exerts bone marrow toxicity that cannot be ignored. Moreover, it may not increase γ-globin expression in splenic erythroid progenitor cells [52,94,116].

Given the limitations of HU, it is essential to develop other drugs, which are summarized in Table 2. Simvastatin, an FDA-approved drug for the treatment of hypercholesterolemia, has also been found to downregulate *BCL11A* expression in laboratory studies and is more effective when combined with tert-butyl hydroquinone than when used alone [117]. Tenofovir disoproxil fumarate has also been shown to downregulate *BCL11A* expression and its trans-acting regulators FOP, KLF1 and GATA1 [118]. It also has a safe clinical history, no cytotoxicity, patient tolerability and oral effectiveness, giving it great advantages as a therapeutic alternative for the development of a new fetal hemoglobin inducer.

In addition, butyrate, isovalerate, isobutyrate, propionate [119], Yisui Shengxue granules [120], 3-phenylisoxazole derivatives [121], benserazide [122], Inula viscosa (a traditional herbal medicine collected from Asinara Island, Italy) [123], pomalidomide [124] and acyclovir have been shown to inhibit the transcription of BCL11A. However, the specific targets of their action are not clear, their safety needs to be effectively evaluated and their mechanisms of action need to be further studied [113].

**Table 2:** BCL11A-related drugs and their mechanisms.

Medicine	Research Progress	Mechanism
Hydroxyurea (HU)	Clinical trial	Downregulating the expression of BCL11A [94,114]
Simvastatin (SIM)	In vitro	Suppressing β-globin mRNA and KLF1 and BCL11A mRNA and protein [117]
T-butylhydroquinone (tBHQ)	In vitro	Suppressing β-globin mRNA and KLF1 and BCL11A mRNA and protein [117]
Romidepsin (ROM)	In vitro	Inhibiting BCL11a expression in combination with SIM [125]
Butyrate, isovalerate, isobutyrate and propionate	In vitro	Repressing the transcription of key gamma-globin regulatory factors, notably BCL11A and SOX6 [119]
Yisui Shengxue granule (YSSXG)	Clinical trial	Promoting the expression of Bcl11a [120]
3-phenyl-isoxazole derivative	Ex vivo	Downregulating Bcl11a and LRF without inhibiting the known epigenetic enzymes [121]
Pomalidomide–nitric oxide (NO) donor derivatives (3a–f)	In vitro	Reducing the level of BCL11A [124]
Inula viscosa (traditional herbal medicine collected from the Island of Asinara, Italy)	In vitro	Downregulating BCL11A expression [123]
Tenofovir disoproxil fumarate (TDF)	In vitro	Downregulating BCL11A and SOX6 and the corresponding trans-acting regulators FOP, KLF1 and GATA1 [118]
siRNA and vincristine (VCR)	In vitro	
Acyclovir	In vitro	Downregulating the mRNA and protein levels of BCL11A [113]

## 11. Conclusions

BCL11A encodes the C2H2 zinc-finger protein, which is highly expressed in the human lymph nodes, thymus and bone marrow tissue but expressed at a low level in most other tissues. As a transcription factor, BCL11A specifically recognizes downstream target genes and constructs transcription or transcriptional coregulators and DNA complexes. Thus, BCL11 plays an important role in regulating various biological processes, especially the activation or inhibition of the hematopoietic system. The transcriptional regulation of BCL11A is implemented throughout the entire cycle of hematopoietic system development and exhibits stage-specific characteristics during hematopoietic lineage differentiation. BCL11A plays an important role in the self-renewal of HSCs and lymphatic maturation. In particular, BCL11A is indispensable for the proliferation, differentiation and survival of B lymphocytes. The mechanism of BCL11A expression in natural killer (NK) cells has rarely been investigated, and, similarly to mature T cells, BCL11A is not expressed in mature NK cells. High levels of BCL11A expression have been detected in NK progenitor cells, suggesting that BCL11A may play a role in them; however, no studies have confirmed this. Different expression levels of BCL11A have been detected in two phenotypically and functionally similar types of pro-NK cells (pre-pro-NKa and pre-pro-NKb), which do not seem to be necessary and can only distinguish between the two cell populations; therefore, whether BCL11A plays an important role in these cells remains to be further investigated [16,126]. In the myeloid system, the most prominent role of BCL11A is as an inhibitory factor for the expression of γ-globin in adult red blood cells and the production of HbF, and it plays a key role in the conversion of human HbF to HbA.

In summary, regarding the role of BCL11A in hematopoietic development, B cells and red blood cells are the most prominent. At the same time, the treatment of blood diseases targeting BCL11A may have other side effects. How to specifically reduce erythroid BCL11A is a direction that needs to be considered in the future.

BCL11A disorders can lead to the abnormal development and function of the hematopoietic lineage, including the myeloid and lymphatic systems, and heterogeneous dendritic cells. Particularly in red blood cells, the destruction of exon 2 of BCL11A can lead to reduced nucleation efficiency. While there is evidence that the loss of BCL11A in CD34+ cells does not affect the expression of several important erythroid transcription factors, such as GATA1 and FOG1, other evidence also supports the idea that BCL11A does not play a prominent role in early erythrocyte development. High expression of BCL11A has been found in various hematopoietic diseases of the lymphatic and myeloid systems and in various types of leukemia. However, studies have shown that acute promyelocytic leukemia is induced by the downregulation of BCL11A expression to inhibit myeloid differentiation, and its specific mechanism requires further study.

BCL11A is an inhibitor of HbF production; the targeted upregulation of HbF expression has become one of the most prominent strategies for the treatment of β-hemoglobin diseases. Various studies have been devoted to knocking down the BCL11A gene, the main targets of which include the erythroid enhancer of BCL11A and exon 2 of BCL11A. The destruction of the BCL11A binding site in the HBG1/2 promoter can also achieve this. Although these strategies are effective in improving HbF expression, there are still many problems, such as low editing efficiency, off-target effects and the potential toxicity of the CRISPR-Cas9 system. The use of multiple editing is currently a prominent trend and can effectively improve the editing efficiency. Gene editing seems to show promising prospects in the treatment of blood diseases related to BCL11A, but how to avoid the possible adverse effects on other cells after editing is worth considering; in particular, some clinical trials have shown serious adverse effects from gene therapy, including blood cancers, liver toxicity and even death [101,127,128]. Moreover, the regulation of BCL11A’s transcription factors, such as the activation of its transcription inhibitors, could be a research direction for disease treatment. BCL11A expression inhibitors, such as SRT2104 and SRT1720, could be combined with HU, which can partially induce HbF. Although many small-molecule compounds can downregulate BCL11A, the mechanisms by which these compounds regulate BCL11A are unclear. Due to the widespread expression of BCL11A, their application may cause side effects. Developing methods that specifically target BCL11A and only act on red line BCL11A will be the focus of future research.

In view of the dominant function of BCL11A in the blood system, further research regarding its role in the development of the hematopoietic system, especially the specific mechanism of action of BCL11A in each cell of the hematopoietic lineage, and the further identification and improvement of upstream and downstream regulatory molecules are warranted. Therapeutic methods targeting BCL11A should also be explored in the future.

## Abbreviations

BCL11A	B-cell lymphoma/leukemia 11A
COUP-TF	Chicken ovalbumin upstream promoter transcription factor
HSCs	Hematopoietic stem cells
GMP	Granulocyte–macrophage progenitors
CLP	Common lymphoid progenitors
AEC	Arterial endothelial cell
SEC	Sinusoidal endothelial cell
VSMC	Vascular smooth muscle cell
Ba	Basophil
Eo	Eosinophil
Ma	Mast cell
RBC	Red blood cell
pDC	Plasmacytoid dendritic cell
MEP	Megakaryocyte erythroid progenitor
MPP	Multipotent progenitor
HSPC	Hematopoietic stem and progenitor cell
Meg/E	Megakaryocyte/erythroid
MSC	Mesenchymal stromal cell
CDP	Dendritic cell progenitor
cDC	Conventional dendritic cell
LSD1	Lysine-specific demethylase 1
SOX6	SRY-Box transcription factor 6
SCD	Sickle cell disease
ALL	Acute lymphoblastic leukemia
NKTCL	Natural killer/T-cell lymphoma
AML	Acute myeloid leukemia
CLL	Chronic lymphocytic leukemia
SLL	Small lymphocytic lymphoma
B-NHL	B-cell non-Hodgkin lymphoma
HD	Hodgkin’s disease
CML	Chronic myeloid leukemia
JMML	Juvenile myelomonocytic leukemia
MLL-r	Mixed lineage leukemia-rearranged
B-ALL	B-cell acute lymphoblastic leukemia
DLBCL	Diffuse large B-cell lymphoma
PML	Promyelocytic leukemia protein
HU	Hydroxyurea
FDA	Food and Drug Administration
SIM	Simvastatin
tBHQ	T-butylhydroquinone
ROM	Romidepsin
YSSXG	Yisui Shengxue granule
NO	Nitric oxide
TDF	Tenofovir disoproxil fumarate
VCR	Vincristine
NK	Natural killer
